# Atlantic Salmon Gill Epithelial Cell Line (ASG-10) as a Suitable Model for Xenobiotic Biotransformation

**DOI:** 10.3390/metabo13060771

**Published:** 2023-06-20

**Authors:** Lada Ivanova, Christiane Kruse Fæste, Anita Solhaug

**Affiliations:** Chemistry and Toxinology Research Group, Norwegian Veterinary Institute, 1433 Ås, Norway; christiane.kruse.faste@vetinst.no (C.K.F.); anita.solhaug@vetinst.no (A.S.)

**Keywords:** aquatic toxicology, ASG-10 epithelial gill cell line, Atlantic salmon, benzocaine, biotransformation, CYP, metabolites, UGT, xenobiotics

## Abstract

Fish are exposed to xenobiotics in the water. Uptake occurs mainly through the gills, which function as an exchange point with the environment. The gills’ ability to detoxify harmful compounds by biotransformation is an essential protection mechanism. The enormous numbers of waterborne xenobiotics requiring ecotoxicological assessment makes it necessary to replace in vivo fish studies with predictive in vitro models. Here, we have characterized the metabolic capacity of the ASG-10 gill epithelial cell line from Atlantic salmon. Inducible CYP1A expression was confirmed by enzymatic assays and immunoblotting. The activities of important cytochrome P450 (CYP) and uridine 5’-diphospho-glucuronosyltransferase (UGT) enzymes were established using specific substrates and metabolite analysis by liquid chromatography (LC) triple quadrupole mass spectrometry (TQMS). Metabolism of the fish anesthetic benzocaine (BZ) in ASG-10 confirmed esterase and acetyl transferase activities through the production of N-acetylbenzocaine (AcBZ), *p*-aminobenzoic acid (PABA) and p-acetaminobenzoic acid (AcPABA). Moreover, we were able to determine hydroxylamine benzocaine (BZOH), benzocaine glucuronide (BZGlcA) and hydroxylamine benzocaine glucuronide (BZ(O)GlcA) by LC high-resolution tandem mass spectrometry (HRMS/MS) fragment pattern analysis for the first time. Comparison to metabolite profiles in hepatic fractions, and in plasma of BZ-euthanized salmon, confirmed the suitability of the ASG-10 cell line for investigating biotransformation in gills.

## 1. Introduction

Aquatic toxicology depends on the availability of reliable models to determine the impact of environmental contaminants on water-living organisms including fish. The assessment of a toxicant’s potential to cause health risks needs to capture molecular properties such as size, stability and lipophilicity, as well as the occurrence and toxicodynamic and toxicokinetic characteristics [1]. The ability of an organism to eliminate a compound by biotransformation and excretion determines if internal concentrations exceed the threshold of toxicological concern and cause toxic effects [2]. Moreover, elimination efficiency is a decisive factor for the extent of bioconcentration after aqueous exposure, leading to increased risks of chronic toxicity and biomagnification at higher trophic levels [3]. Fish are exposed to a multitude of xenobiotics, non-physiological chemical substances such as pharmaceuticals and environmental toxins, through the diet and the marine environment. 

The uptake of waterborne chemicals occurs primarily via gills that permit efficient exchange to the blood through a thin membrane with a large surface area [4]. The diffusion of a compound across the lamellar epithelium is affected by its protein binding affinity and lipid solubility, which often is described by the n-octanol-water partition coefficient (pK_OW_). In general, gill uptake is inefficient for molecules with a pK_OW_ below 1 and increases four-fold up to pK_OW_ 3, which applies to many aquatic contaminants and fish pharmaceuticals, leading to considerable trans-gill absorption rates [5]. The local anesthetic benzocaine (BZ) (ethyl 4-aminobenzoate), commonly used for fish sedation in aquaculture, belongs to this group [6]. Under water exposure, BZ reaches maximal concentrations in blood in less than 2 min, demonstrating high gill transition and efficient uptake [7,8]. The drug is metabolized in fish by hepatic biotransformation to three known metabolites and has a plasma half-life of less than 2 h in salmonids. 

In the present study, we have used BZ as a model compound to evaluate the capacity of the Atlantic salmon (*Salmo salar*) gill epithelial cell line ASG-10 [9] to emulate in vivo metabolite production in salmon gills. Furthermore, the activities of major biotransformation enzymes, responsible for oxidoreductive and conjugative metabolism reactions, were characterized to establish the model’s functionalities. Atlantic salmon is extensively farmed worldwide, with Norway as the greatest producer. Gill-related diseases from waterborne agents are a major problem in salmon aquaculture [10]. Currently, etiological studies primarily involve live fish, although in vitro assays are increasingly utilized to replace, reduce, and refine (3R) in vivo trials. [11]. The sheer number of environmental contaminants that have to be assessed for risk in an ecotoxicological context requires a departure from mass testing in fish and a shift to more sustainable methods. Two strategies are usually followed: cell-based in vitro assays are predominantly performed to establish the cytotoxicity of selected compounds, whereas incubations with liver fractions are used to study biotransformation and bioaccumulation potential [12]. Accordingly, gill epithelial cells have been used as a toxicity screening tool [13] and to determine uptake rates [14]. One report describing metabolism experiments with the gill cell lines RTgill-W1 of rainbow trout (*Oncorhynchus mykiss*) and G1B of walking catfish (*Clarias batrachus*) did not find relevant biotransformation activity, not even of cytochrome P450 1A1 (CYP1A1), the most important metabolizing enzyme in gills in vivo [15].

In this context, it was the aim of the present study to establish a functioning model for xenobiotic biotransformation in gill cells that would allow the in vitro-to-in vivo prediction of metabolites formed from marine contaminants. Regarding the importance of Atlantic salmon in Norwegian aquaculture, we chose the Atlantic salmon gill epithelial cell line ASG-10 as a model and characterized individual enzyme activities and BZ metabolite profiles, comparing the findings to results observed in vivo and in hepatic fractions. 

## 2. Materials and Methods

### 2.1. Chemicals and Reagents

Leibowitz’s L-15 Glutamax cell culture medium, Dulbecco’s Modified Eagle Medium (DMEM) w/o phenol red, fetal bovine serum (FBS) (USA origin), penicillin/streptomycin, trypsin (trypLE) and β-merceptaethanol were obtained from Gibco (Thermo Fisher; Waltan, MA, USA). The MycoAlert^®^ Mycoplasma detection kit was purchased from Lonza (Basel, Switzerland), while cell lysis buffer (#9803), nonfat dry milk and horseradish peroxidase (HRP)-conjugated anti-mouse antibody were obtained from Cell Signaling Tec (Beverly, MA, USA). Proteinase inhibitor cocktail (P8340), β-naphthoflavone (BNF), dimethylsulfoxide (DMSO), 7-ethoxy-resorufin, Tween 20 and sodium dodecyl sulfate (SDS) were obtained from Sigma-Aldrich (St. Louis, MO, USA). The rapid cell homogenization device QIAshredder was obtained from Qiagen (Hilden, Germany), and the Bio-Rad DC kit for total protein content determination was obtained from Bio-Rad Laboratories Inc. (Hercules, CA, USA). The chemoluminescent substrate SuperSignal West pico plus and Alamar Blue were purchased from Thermo Scientific. CYP1A (fish) monoclonal antibody C10-7 was obtained from Cayman Chemical (Ann Abor, MI, USA), and CelltoxGreen was obtained from Promega (Madison, WI, USA). NuPage reagents for immunoblotting were all purchased from Thermo Fisher, Invitrogen. Optima LC−MS grade water (H_2_O), acetonitrile (MeCN), isopropanol (IPA) and methanol (MeOH) were provided by Fisher Scientific (Oslo, Norway). Benzocaine (BZ), *p*-acetylamino benzoic acid (AcPABA), 4-aminobenzoic acid (PABA), acetylbenzocaine (AcBZ), phenacetin (PCN), acetaminophen (ACP), tolbutamide (TB), 4-hydroxytolbutamide (4-OH-TB), dextromethorphan (DEX), dextrophan (DOR), chlorzoxazone (CH), 6-hydroxychlorzoxazone (6-OH-CH), midazolam (MDZ), 4-hydroxymidazolam (4-OH-MDZ), β-estradiol (E2), β-estradiol 17-(β-D-glucuronide) sodium salt (E2-GlcA), N-acetyl serotonin (NAS), naloxone (NLX), naloxone-3β-D glucuronide solution (NLX-GlcA), mycophenolic acid (MA), mycophenolic acid-β-D-glucuronide (MA-GlcA), trifluoperazine dihydrochloride (TFP) and trifluoperazine N-β-D-glucuronide (TFP-GlcA) were purchased from Sigma-Aldrich. N-acetyl serotonin β-D-glucuronide (NAS-GlcA) was obtained from Santa Cruz Biotechnology (Dallas, TX, USA). Buffer and substances used for the in vitro metabolism experiments, including β-nicotinamide adenine dinucleotide phosphate sodium salt (NADP^+^), β-nicotinamide adenine dinucleotide phosphate reduced tetrasodium salt (NADPH), D-glucose-6-phosphate sodium salt, D-glucose-6-phosphate dehydrogenase from Baker’s yeast (*Saccharomyces cerevisiae*), uridine 5′-diphosphoglucuronic acid tri-sodium salt (UDPGA), uridine 5′-diphospho-N-acetylglucosamine sodium salt (UDPAG), MgSO_4_, KH_2_PO_4_ and HEPES buffer, were obtained from Sigma-Aldrich. UDP-glucuronosyltransferase (UGT) Reaction Solution B containing 250 mM Tris-HCl, 40 mM magnesium chloride (MgCl_2_ × 6H_2_O) and 0.125 mg/mL alamethicin in water was obtained from BD Biosciences (Woburn, MA, USA).

### 2.2. Cell Culture

The ASG-10 cell line was developed at the Norwegian Veterinary Institute [9]. The cells are grown in Leibowitz’s L-15 Glutamax medium supplemented with 10% FBS, 1% penicillin/streptomycin and 30 µM β-merceptaethanol at 19 °C in a non-ventilated cell culture flask in a tempered incubator and sub-cultured 1:2 every 10 days following detachment using trypLE. The cell lines are routinely checked for mycoplasma infection using the MycoAlert^®^ Mycoplasma detection kit. 

The ASG-10 cells were plated out two days before the exposure experiments at a concentration of 132,000/cm^2^ using complete cell culture medium without β-merceptaethanol. The cells were 100% confluent at the day of exposure.

### 2.3. Cell Viability

Alamar Blue assay: The metabolic activity/viability of the ASG-10 cell line was measured using the Alamar Blue assay according to the manufacturer’s protocol (Thermo Fisher). After exposure to test compounds with potential cell toxicity, Alamar Blue was added to the cell culture, and incubated for 3 h. The dark blue oxidized form of Alamar Blue is enzymatically reduced by living cells to a highly fluorescent form, and the measured fluorescence intensity is thus proportional to the number of viable cells. The fluorescence (excitation: 555 nm/emission: 585 nm) was quantified using a Spectramax i3x plate reader (Molecular Devices, San Jose, CA, USA). 

CellTox™ Green assay: The non-toxic dye CellTox™ Green stains DNA in cells with impaired membrane integrity. Binding interactions with DNA produce a fluorescence signal that is proportional to cytotoxicity (necrotic, late apoptotic cells). CellTox™ Green dye was added to the cells as described by the manufacturer (Promega) and fluorescence visualized by microscopy (Zeiss Observer A1, Carl Zeiss, Oberkochen, Germany).

### 2.4. Cytochrome P450 (CYP) 1A Immunochemical Analysis 

The ASG-10 cells were seeded into 6-well plates as described in Section 2.2. After two days, the cells were stimulated with BNF (1–100 nM, 24 h), a well-known non-toxic inducer of CYP1A protein expression [16]. After exposure, the cells were washed twice in ice-cold PBS and placed at −70 °C until the next day. The cells were then scraped in cell lysis buffer with 0.8% SDS and 1% proteinase inhibitor cocktail added and centrifuged (10,000× *g*, 1 min) through use of the QIAshredder for homogenization. The protein concentrations were quantified by using the Bio-Rad DC kit (Hercules, CA, USA) and the samples were adjusted to equal protein concentration with lysis buffer before being denatured through the addition of NuPage lithium dodecyl sulfate (LDS) sample buffer and NuPage reducing agent as described by the manufacturer (Invitrogen, Waltham, MA, USA). Blotting was performed using the NuPage Novex system from Invitrogen (Thermo Fisher), applying 30 ng protein per well on a 4–12% NuPage BisTris gel. Separation was performed under reducing conditions for 40 min at 200 V in 3-(N-morpholino) propane sulfonic acid (MOPS)–SDS running buffer using the Novex Sharp pre-stained standard with a range of 3.5–260 kDa. Proteins were electrophoretically transferred from the gel onto a nitrocellulose membrane (Bio-Rad, Hercules, CA, USA) for 60 min at 30 V with transfer buffer in an XCell II Blot Module (Invitrogen). The membranes were blocked in 5% milk in Tris-buffered saline (TBS, pH 7.6) for 60 min at room temperature and washed twice for 10 min in TBS containing 0.05% Tween 20 (TBST, pH 7.6). For the detection of piscine CYP1A, the monoclonal antibody C10-7 (1:500) was used as the primary antibody, which was diluted in 1% milk in TBST and incubated at 4 °C overnight, and HRP-conjugated anti-mouse antibody (1:500) was used as a secondary antibody, which was diluted in TBST and incubated for 1 h at room temperature. CYP1A protein expression was visualized by chemiluminescence with SuperSignal West pico plus as substrate, measuring signal intensities in the Chemidoc XRS^+^ imaging system (BioRad, Hercules, CA, USA).

### 2.5. EROD Assay

CYP1A induction in BNF-stimulated ASG-10 was measured using the ethoxyresorufin-O-deethylase (EROD) assay. CYP1A converts 7-ethoxyresorufin to resorufin, which can be measured by fluorescence spectroscopy [16]. The assay was performed as described in [17], with minor modifications. Briefly, the cells were seeded in a black 96-well plate as described in Section 2.2. After two days, the cells were incubated with the CYP1A inducer β-naphthoflavone (BNF; 1–100 nM) for 24 h. BNF was dissolved in DMSO. The final DMSO concentration in the cell culture was 0.1%. Appropriate controls containing the same amount of DMSO were included in each experiment. The next day, the culture medium was replaced with 200 µL EROD assay media (DMEM w/o phenol red; 10% FBS, 8 µM 7-ethoxyresorufin), and resorufin fluorescence (excitation: 530 nm/emission: 580 nm) was detected after 60 min using a Spectramax i3x plate reader.

### 2.6. Preparation of S9 Fractions and Liver Microsomes from Atlantic Salmon

Five one-year-old salmon (NMBU, Ås, Norway) were sacrificed by a blow to the head, and the livers (*n* = 5; total weight = 21.6 g) were extracted, washed with ice-cold physiological saline and stored at −80 °C until further use. Liver microsomes from Atlantic salmon were prepared by differential centrifugation following an established protocol [18]. The livers were cut and homogenized on ice in 0.1 M potassium phosphate buffer (pH 7.5) with a Potter-Elvehjem homogenizer (Sigma-Aldrich). The homogenate was centrifuged at 9000× *g* for 20 min at 4 °C (Beckman Instruments, Palo Alto, CA, USA). Part of the resulting supernatant was collected as S9 and transferred to a clean tube. The remaining supernatant was used to prepare salmon liver microsomes by centrifugation at 100,000× *g* for 60 min at 4 °C using the swing-out rotor TH-641 (Beckman). The pellet was resuspended and homogenized in 0.1 M potassium buffer. All processing steps were carried out on ice. The prepared salmon liver microsomes (SLMs) and the S9 fraction were stored in aliquots at −80 °C until use. Total protein was determined using the Bio-Rad DC kit.

### 2.7. Preparation of Chemical Standards

Enzyme activities in the ASG-10 cells and hepatic fractions were characterized by using specific substrates and detecting their respective metabolism products. The substrate concentrations used in the assays are summarized in Table 1. Substrate stock solutions (1 mg/mL) were prepared in MeOH, except for MA-GlcA and NLX-GlcA that were solved in MeCN and MeOH:H_2_O (9:1 *v*/*v*), respectively. The combined substrate solutions C1 to C4 were prepared in 100% MeOH. CYP and uridine 5’-diphospho-glucuronosyltransferas (UGT) enzyme activities in the hepatic fractions were determined by using solutions C2 and C4, respectively. In the ASG-10 cells, C1 and C3 were used to measure the activities of the biotransformation enzymes. The solutions were diluted in incubation media at the day of the experiment, obtaining assays concentrations of 2, 5 and 10 µM of the different substrates (Table 1).

Stock solutions (1 mg/mL) of BZ (6.1 µM) and BZ metabolites (AcPABA, 5.6 µM; PABA, 7.3 µM; AcBZ, 4.8 µM) were prepared in 100% MeOH and stored at −20 °C. The working solutions W6 and W7 were prepared in 100% MeOH. For the metabolism assays in ASG-10 cells, the BZ working solution (W5) was diluted with incubation medium on the day of the experiment. The final MeOH concentration in the cell culture was a maximum of 1.0%. Appropriate controls containing the same solvent levels were included in all experiments.

### 2.8. Characterization of Biotransformation Capacities

CYP activities in ASG-10, SLMs and S9 were determined by using a mixture of the specific substrates PCN (CYP1A2), TB (CYP2C9), DEX (CYP2D6), CH (CYP2E1) and MDZ (CYP3A4) (Table 1), as previously described [1,2]. Likewise, UGT activities were determined by incubation with the specific substrates E2 (UGT1A1), TFP (UGT1A4), NAS (UGT1A6), MA (UGT1A9) and NLX (UGT2B7). The production of the respective metabolites was measured by liquid chromatography triple quadrupole mass spectrometry (LC-TQMS). The different CYP and UGT substrates were semi-quantified based on measured peak areas, whereas their respective main metabolites were semi-quantified using matrix-assisted calibration curves (Supplementary Tables S1 and S2). The metabolites were determined by comparison of their retention times and mass spectra to those of reference standards. 

BZ, an anesthetic commonly used in aquaculture, was used to evaluate the potential activity of N-acetyltransferases (NAT) and esterases in ASG-10, SLMs and S9. The production of BZ-related metabolites was determined by liquid chromatography high-resolution tandem mass spectrometry (LC-HRMS/MS). When available, reference standards were used for the specification of metabolites.

#### 2.8.1. ASG-10

The cells were plated in a 96-well plate as described in Section 2.2. The combined substrate solutions C1 or C3 were diluted in culture medium and 100 µL was added to each well, which already contained 100 µL culture medium, thus reaching the final assay concentrations (Table 1). After incubation for 24 h at 19 °C, 75 µL of medium was removed and mixed with an equal volume of ice-cold 100% MeCN. Substrate and metabolite concentrations were determined by LC-TQMS. 

BZ metabolism was investigated in ASG-10 cells plated in a 96-well plate as described in Section 2.2. The culture medium was replaced by fresh medium containing BZ (Table 1). After incubation for 24 h at 19 °C, 75 µL of medium was removed, mixed with the same volume of ice-cold 100% MeCN and analyzed by LC-HRMS/MS for BZ and metabolite content.

#### 2.8.2. SLMs and S9

CYP activities in SLMs (2 mg/mL protein) and S9 (4 mg/mL protein) were investigated by incubating C2 (Table 1) at 20 °C in a shaking water bath (OLS 200; Grant, Cambridge, UK) in 1 mL 0.05 M HEPES buffer (pH 7.4) containing NADPH, NADP^+^, glucose 6-phosphate, MgCl_2_ × 6H_2_O and 1 U/mL glucose-6-phosphate dehydrogenase. The cofactor concentrations used for SLMs and S9 are shown in Table 2. After preincubation for 3 min, the reaction was started by addition of 3.5 µL of C2. Incubation aliquots (120 µL) were drawn after 0, 5, 10, 15, 30 and 60 min and mixed on ice with the same volume of ice-cold 100% MeCN. After vortexing and centrifugation at 20,000× *g* for 10 min at 4 °C, the samples were analyzed by LC-HRMS/MS. Metabolite concentrations were determined by using matrix-assisted calibration curves (7.5 ng/mL to 300 ng/mL) in incubation medium/MeCN (50:50). 

UGT activities in SLMs (2 mg/mL) and S9 (4 mg/mL) were determined using C4 (Table 1) in 0.5 mL of incubation mixture containing 7.5 mM UDPGA, 0.3 mM UDPAG and Reaction Solution B (1:5). After pre-incubation at 20 °C for 2 min, 3.5 µL of C4 was added to start the reaction. Incubation aliquots (120 µL) were drawn at 0, 5, 10, 15, 30 and 60 min and mixed with the same volume of ice-cold 100% MeCN. Samples were centrifuged at 20,000× *g* for 10 min at 4 °C, and the supernatants were analyzed by LC-TQMS. 

Additionally, BZ metabolism was investigated in SLMs (1 µM BZ) and S9 (3 µM BZ) (Table 1). The incubations were first performed separately for phase I and phase II metabolism, and a combined assay was performed afterwards. The reaction mixture (total assay volume 0.5 mL) contained 0.8 mM NADPH, 0.7 mM NADP+, 7.5 mM UDPGA and 0.3 mM UDPAG. Aliquots (150 µL) were drawn at 0, 30 and 60 min and mixed with an equal volume of ice-cold 100% MeCN. In order to increase measurable metabolite levels, the 30 min and 60 min incubation aliquots were combined, evaporated to dryness using a gentle stream of nitrogen at 40 °C and reconstituted in 70 μL MeCN/water (50:50). After filtration (Costar Spin-X^®^ 0.22 mm Nylon filter; Corning, Inc.; Corning, NY, USA), the concentrated samples were analyzed using LC-HRMS/MS. 

### 2.9. Plasma Extraction

Plasma collected from the caudal vein of on-growing Atlantic salmon that belonged to an untreated control group of a different study was acquired because the fish had been euthanized with a BZ overdose (200 mg/L). Subsequently, 100 µL of heparinized plasma was mixed with 300 µL ice-cold MeOH, vortexed for 20 sec and centrifuged at 20,000× *g* for 10 min at 4 °C. The supernatants of three replicates were pooled together, transferred into a 2 mL glass tube and dried by nitrogen stream at 40 °C. The residues were re-dissolved into 120 µL 50% MeCN and filtrated (Costar Spin-X^®^) before LC-HRMS/MS analysis. 

### 2.10. Protection of Free Amino Groups from Hydroxylation by Acetylation

A volume of 30 µL of 1 mg/mL BZ in 100% MeOH was evaporated (40 °C, N_2_) in a 1.5 mL vial. The residue was redissolved in 100 µL acetic anhydride (Sigma) and 100 µL pyridine (Sigma). The vial was placed at room temperature for 18 h. Subsequently, the reaction was stopped by the addition of 200 µL 100% MeOH. After evaporation, the residue was dissolved in 150 µL 100% MeOH (solution A). As control, 30 µL of BZ solution was evaporated and re-dissolved in 150 µL 100% MeOH (solution B). Afterwards, 3 µL of solutions A and B were incubated in SLMs as described above. 

### 2.11. Mass Spectrometric Analysis Using Triple Quadrupole (TQMS) and High-Resolution Tandem Mass Spectrometry (HRMS/MS) Systems

LC-TQMS analyses of CYP and UGT substrates and formed metabolites were performed as described by Johny et al. [18]. The LC-TQMS consisted of an Agilent 1290 Infinity Binary UHPLC System with a vacuum degasser and column maintained at 30 °C, which interfaced with an electrospray ionization source with an Agilent 6470 triple quadrupole mass spectrometer (Agilent Technologies, Santa Clara, CA, USA). Mass spectral data acquisition was achieved under simultaneous polarity switching. Dynamic multiple reaction monitoring (MRM) was used to collect at least two precursor ion-to-product ion transitions (qualifier and quantifier) per analyte. Ion transitions were registered simultaneously in positive and negative ion mode. CYP substrates and their respective metabolites was determined using integral MS parameters as follows: ESI capillary voltage 2.5 kV/−2.5 kV (ESI+/−, respectively), nebulizer gas (N_2_) pressure 35 psi, dry gas temperature 300 °C with a flow rate of 8.0 L/min, sheath gas temperature 350 °C with a flow rate of 11.0 L/min. For the determination of UGT substrates and their metabolites, the following parameters were applied: ESI capillary voltage 4.0 kV/−3.5 kV (ESI+/−, respectively), nozzle voltage 500/−1.0 kV (ESI+/−, respectively), nebulizer gas (N_2_) pressure 35 psi, dry gas temperature 300 °C with a flow rate of 10.0 L/min, sheath gas temperature 375 °C with a flow rate of 12.0 L/min. Chromatography was performed using an a 150 × 2.1 mm i.d. Kinetex F5 2.6 μm UHPLC column with a 0.5 μm × 0.004 in i.d. KrudKatcher Ultra Column in-line filter (both Phenomenex). CYP substrates and metabolites (injection volume 1 µL) were eluted using a binary gradient consisting of solvent A (0.1% formic acid in water) and solvent B (0.1% formic acid in MeCN) at a flow rate of 0.25 mL/min, starting at 18% B for 1 min and increasing to 55% B in 12 min and 95% in 1 min. After maintaining 95% B for 3 min, the column was re-equilibrated with 18% B for 4 min within a total run time of 21 min. UGT substrates and metabolites were separated with a slightly different binary gradient starting at 2% B for 1.5 min, increasing to 39% B at 4 min, to 55% B at 10.5 min and to 95% B at 11 min. After washing with 95% B for 2 min, the mobile phase was returned to the initial conditions and the column re-equilibrated for 2 min, with a total run time of 15 min. Data acquired by TQMS were analyzed with MassHunter™.

BZ biotransformation products were determined using a Q Exactive™ hybrid quadrupole-Orbitrap mass spectrometer equipped with a heated electrospray ion source (HESI-II) and coupled to a Vanquish UHPLC system (Thermo Fisher Scientific). The HESI-II interface was operated in positive ion mode at 300 °C; other parameters were adjusted as follows: spray voltage 3.2 kV, heated transfer capillary temperature 280 °C, sheath gas flow rate 35 L/min, auxiliary gas flow rate 10 L/min, S-lens RF level 55.

Mass spectra were acquired in the full scan mode, applying sequential data-dependent top 5 analysis (ddMS2-top5) with an inclusion list containing masses of interest. The settings for full scan acquisition were as follows: resolution of 70,000 full width at half maximum (FWHM); automatic gain control (AGC) target 1 × 10^6^; maximum injection time (IT) 100 ms; scan range *m*/*z* 170–600. MS/MS fragmentation spectra were acquired at 17,500 FWHM. The intensity threshold to trigger MS/MS acquisition was 2.0 × 10^5^. Fragmentation analysis was carried out using a higher energy collisional dissociation (HCD) cell with stepped normalized collision energy (NCE) at 20, 40 and 60 eV. The settings for the AGC target, IT and isolation window were set to 5 × 10^4^, 150 ms and 1.8 *m*/*z*, respectively. BZ and BZ-related compounds were determined by targeted parallel reaction monitoring (PRM). The PRM parameters were set to the following: mass resolution, 70,000; AGC target, 2 × 10^5^; maximum IT, 100 ms; *m*/*z* isolation window within 1.8. The maximum mass accuracy shift was limited to 5 ppm. 

The mobile phase was composed of solvent A (water) and solvent B (MeOH), both containing 0.1% formic acid. Chromatographic separation was achieved on a Thermo Scientific™ Hypersil Gold™ aQ Polar Endcapped C18 column (100 mm × 2.1 mm, 1.9 µm) at a flow rate of 0.4 mL/min. After sample injection (2 µL), elution started with 0% B for 1 min, followed by 5% B from 1 to 5 min, a linear increase to 95% from 5 to 10 min and continued elution at 95% B from 10 to 13 min. The column was re-equilibrated within 2.5 min to 0% B before each analysis. The temperature of the column oven and autosampler tray was set at 55 °C and 10 °C, respectively. Raw data were acquired by Xcalibur (Version 4.0, Thermo Fisher Scientific), and molecular formula assignments and mass error were evaluated within the same software (Elemental Composition tab). Extracted ion chromatograms were obtained with a mass error of ±5 ppm.

### 2.12. Statistical Analysis

The data analyses were performed using GraphPad Prism version 9.0.1 (151). Statistical significance (*p* < 0.05) was assessed by one-way-ANOVA, followed by Dunnett’s post-test.

## 3. Results and Discussion

The gills are a major point of entry for waterborne chemicals in fish, leading to direct uptake into the systemic circulation [4]. Consequently, high blood concentrations might be reached since the intestinal uptake and the hepatic first pass are bypassed. The capability of the gill epithelial cells for xenobiotic biotransformation is thus of considerable importance for protecting fish against harmful environmental substances [5]. In the present study, we have characterized the biotransformation activities of the epithelial gill cell line ASG-10 by using typical substrates of CYP and UGT enzymes, as well as the fish anesthetic BZ. In addition to LC-TQMS, which is commonly used for quantifying known compounds, HRMS was employed to acquire full-scan spectra, facilitating the detection of unknown metabolites in biological samples. HRMS offers the advantage of providing additional structural information about unknown compounds through the comparison of accurate mass data and fragmentation patterns with known reference standards. The produced metabolites were compared to results obtained in hepatic fractions (SLMs and S9) and in the plasma of BZ-exposed salmon. 

### 3.1. CYP Activities in ASG-10

Fish contain 18 CYP gene superfamilies, including CYP1 to CYP4, which are mainly involved in xenobiotics metabolism [19,20]. The enzymes typically catalyze monooxygenase reactions, converting substrates into metabolites with increased hydrophilicity that are less toxic and more easily excreted from the organism [21]. The majority of CYP enzymes characterized in marine and freshwater fish have been determined in the liver, the main metabolism organ, but there are also reports on CYP in the gills and intestine [22,23,24].

CYP1A plays a prominent role in the biotransformation of environmental pollutants in different fish species, including Atlantic salmon [25]. It is inducible by aryl hydrocarbons such as BNF, phenanthrene and oiled sediments [26,27,28], while expression is suppressed by the steroid 17ß-estradiol [29,30]. Because of these regulation mechanisms, piscine CYP1A levels are used as diagnostic and predictive biomarkers for pollution monitoring in aquatic environments [31,32,33]. 

**Figure 1:**
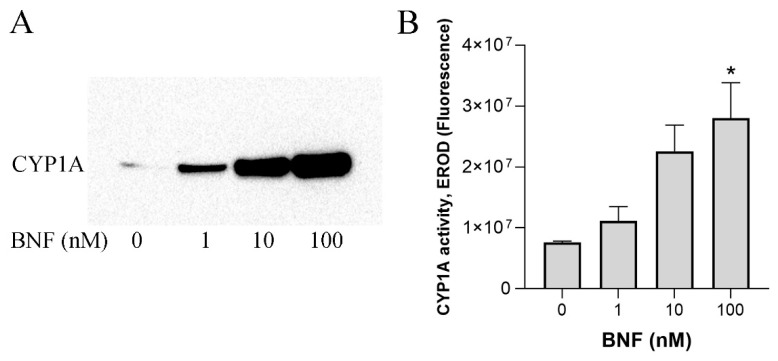
CYP1A induction in ASG-10 after incubation with BNF (1 to 100 nM, 24 h), detected by (**A**) expressed CYP1A protein, as shown by immunochemical analysis, and (**B**) CYP1A enzyme activity, as measured in the EROD assay. The data represent the means ± SD of three independent experiments, each with three technical replicates. Significant differences (*p* < 0.05) as compared to the control (0 µM) are indicated with an asterisk (*).

Considering the importance of CYP1A, we started the characterization of ASG-10 by investigating the inducibility of activity by BNF exposure. CYP1A protein levels were determined by immunochemical analysis (Figure 1A), and CYP1A enzyme activity was measured using the standardized EROD assay, which is commonly used in aquatic biomonitoring (Figure 1B) [32,34]. The results of the two assays were mutually confirmative, showing a BNF concentration-dependent increase in both the CYP1A protein and activity after incubation for 24 h.

ASG-10 already demonstrated high sensitivity to BNF at sub-micromolar concentrations. Exposure to 1 nM BNF for 24 h resulted in a visible CYP1A protein increase in the immunochemical analysis, showing that the gill cells contained inducible CYP1A1, comparable to live fish [25]. In contrast, the responsiveness of a clearfin livebearer (*Poeciliopsis lucida*) hepatocellular carcinoma cell line (PLHC-1) was lower [35], requiring induction with 1 µM BNF for 24 h to produce a detectable CYP1A protein increase. In the EROD assay, PLHC-1 showed an eightfold increase in enzyme activity after stimulation with 100 nM BNF, which increased to 100-fold with 1 µM BNF. In cultured primary hepatocytes of carp (*Cyprinus carpio*), exposure to 20 nM for 18 h led to a 30-fold increase in the EROD rate [36]. A primary cell culture from rainbow trout (*Oncorhynchus mykiss*) liver showed a dose-dependent EROD rate increase (maximum nine-fold) after exposure to 3.6 to 360 nM BNF for 48 h [37], which is comparable to the results observed for ASG-10 in the present study and the gill cell line (LG-1) of Atlantic lumpfish (*Cyclopterus lumpus*) [17]. In contrast, CYP1A activity was not detectable in the gill cell lines RTgill-W1 of rainbow trout and G1B of walking catfish [15].

**Figure 2:**
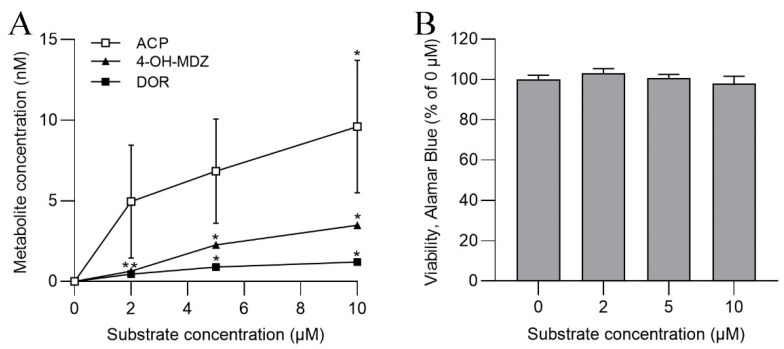
(**A**) Formation of metabolites of specific CYP substrates in ASG-10 after incubation for 24 h. (**B**) Cell viability in the presence of CYP substrates, determined in the Alamar Blue assay. The data represent the means ± SD of three independent experiments, each with three technical replicates. Significant differences (*p* < 0.05) as compared to the control (0 µM) are indicated by asterisk (*).

After establishing inducible CYP1A metabolism in ASG-10 in dedicated assays, we used a broadened approach for the determination of major CYP reactivities in non-induced cells. Using substrates that are specific to five major human CYPs belonging to the CYP1, CYP2 and CYP3 superfamilies (Table 1), the formation of the respective metabolites in ASG-10 after incubation for 24 h was measured (Figure 2A). We found considerable enzymatic activity congruent to reactions catalyzed by salmon orthologs of human CYP1A2, CYP2D6 and CYP3A4, whereas activity comparable to CYP2C9 and CYP2E1 reactivity was not discernible. ACP, the product of PCN by CYP1A-related metabolism, showed the highest formation rate. The CYP450 substrates did not affect overall ASG-10 viability as measured in the Alamar Blue assay (Figure 2B).

Biotransformation enzymes in gills have not been studied in much detail so far in spite of their importance for the defense of fish against environmental toxicants. In the gills of coho salmon (*Oncorhynchus kisutch*) [23], CYP1A1-catalyzed EROD activity was found at a low level, but metabolism capacity related to CYP2M1, CYP2K1 and CYP3A27, the equivalent of mammalian CYP3A4, was not measurable. Surprisingly, the CYP isoform expression in the gills was determined as CYP3A27 > CYP2M1 > CYP1A1 [23]. S9 prepared from rainbow trout and channel catfish (*Ictalurus punctatus*) gills had EROD activity and was additionally capable of metabolizing typical CYP2D6, CYP2C9 and CYP3A4 substrates [3]. The metabolic rates were highest for CYP1A1 and CYP2D6 and rather low for CYP3A4, comparable to our results in ASG-10. In a follow-up study, we studied the expression of CYP and UGT genes in ASG-10 and the primary gill tissue of Atlantic salmon parr [38]. While the expression levels differed for individual enzymes, enzyme composition was generally the same in both materials, showing the presence of important enzymes such as CYP1A1, CYPM1, CYP3A27 and UGT1A1. 

### 3.2. UGT Activities in ASG-10

Metabolism by conjugation reactions (phase II) is as equally important a detoxification mechanism as metabolism by oxidoreductive metabolic reactions (phase I). After defining the CYP enzyme profile in ASG-10, we therefore continued characterizing the metabolic capacity of the gill cell line with regard to its UGT enzyme activities. Information on functional piscine UGT is limited, although teleost fish have considerably more UGT genes than mammals [39]. In zebrafish (*Danio rerio*), 40 genes encoding UGT were identified, belonging to the UGT1, UGT2 and UGT5 families [39]. Since orthologues to mammalian UGT have not been identified at the protein level, prediction of catalytic specificities is difficult. Nevertheless, the applicability of specific substrates (Table 1) of human UGT to salmon isoforms has been shown recently [18].

**Figure 3:**
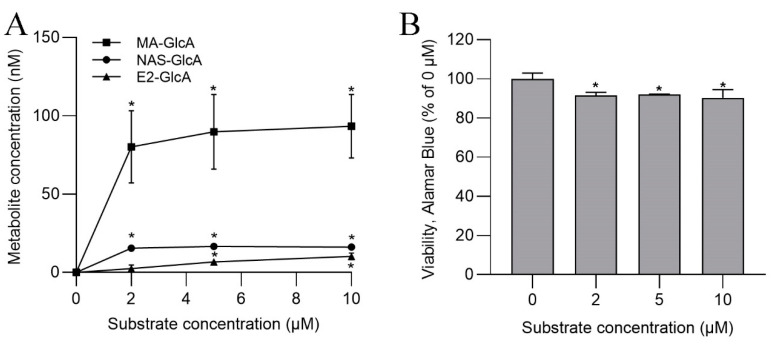
(**A**) Formation of UGT-specific metabolites in ASG-10 after incubation for 24 h. (**B**). Cell viability in the presence of UGT substrates, as determined in the Alamar Blue assay. The data represent the means ± SD of three independent experiments, each with three technical replicates. Significant differences (*p* < 0.05) as compared to the control (0 µM) are indicated by an asterisk (*).

The incubation of ASG-10 with the C3 solution resulted in the formation of several glucuronides (Figure 3A). MA-GlcA, the product of MA and human UGT1A9, reached the highest concentrations (up to 117 µM). Furthermore, significant activities related to UGT1A1 and UGT1A6 were determined, whereas glucuronidation catalyzed by UGT1A4- and UGT2B7-like enzymes was not detected. The UGT substrates had only a minor effect on the metabolic activity/cell viability measured in the Alamar Blue assay (Figure 3B). 

Considering the notable species differences, direct comparison of the UGT activities determined in ASG-10 with data obtained for other fish or mammals is not unproblematic. However, among the UGT genes identified in the zebrafish genome with tissue-specific expression profiles [39], several belonged to the UGT1A, UGT1B, UGT2A and UGT2B families [39]. They showed glucuronidation activity toward ten typical substrates. UGT1A1, UGT1A7 and UGT1B1 had especially high affinity toward phenolic aglycones such as bisphenol A, 4-nitrophenol and 1-naphthol, and carboxylic acids, e.g., diclofenac. Members of the UGT5 family, which exists in teleosts and amphibians but is not present in mammals, showed a high specificity to steroid hormones, including E2 [39]. Comparative analyses revealed that the A and B clusters of UGT1 and UGT2 have orthologs in other fish species [40]. Functional assays demonstrating glucuronidation of phenolic compounds and steroids in liver microsomes of plaice (*Pleuronectes platessa*) [41] have confirmed this conformity. Moreover, gene analyses in the liver, gills, kidneys, fat and muscles of plaice, flounder (*Platichthys flesus*) and pufferfish (*Tetraodon nigroviridis*) have determined homologies between fish UGT genes and considerable similarities to some human UGT1 and UGT2 isoforms [42].

The UGT characterization in ASG-10 in the present study revealed a similar activity pattern to that in zebrafish, indicating the presence of proteins related to UGT1A1, UGT1A6 and UGT5E1. UGT in Atlantic salmon (taxonomy_id:8028; UniProt.org; accessed on 15 March 2023) has not been identified at protein or transcript levels but is derived from genome information by homologies to orthologs in related species. In zebrafish, tissue-specific gene expression analysis showed elevated levels of UGT1A, UGT2A, UGT5B, UGT5E, UGT5F and UGT5G in the gills, assigning these enzymes a considerable role in xenobiotic biotransformation [39]. Thus, the conservation of this functionality in ASG-10 underlines their applicability for in vitro metabolism studies of environmental pollutants. 

Gill cell culture research has been intensified in the last 20 years, especially with regard to aquatic environmental monitoring [43]; however, the ASG-10 cells present, to our knowledge, the first gill cell system that can be considered as a suitable model for xenobiotic biotransformation, producing reproducible and reliable results. Moreover, ASG-10 is the first gill cell line derived from Atlantic salmon, for which previously only cell lines derived from macrophages (SHK-1), head kidney (TO) and fibroblasts (AS) and models with primary cells existed. As the next step, we therefore continued to elucidate the metabolic potential of ASG-10 by comparing the measured CYP and UGT profiles to those in the hepatic fractions of SLMs and S9. 

### 3.3. CYP Activities in SLM and S9

Both hepatic fractions, prepared from fish that had not been exposed to any drug or anesthetic, were incubated with the same CYP substrates used for ASG-10 (Table 1). Liver microsomes (SLMs) are obtained from the endoplasmatic reticulum of hepatocytes and contain membrane-bound metabolism enzymes (e.g., CYP and UGT), whereas the hepatocyte S9 fraction also includes cytosolic enzymes and is more similar to the intact cells, only lacking cellular organization. Comparing incubation results obtained by both fractions thus provides an indication regarding the involvement of soluble enzymes in the biotransformation pathway of a substance. 

**Figure 4:**
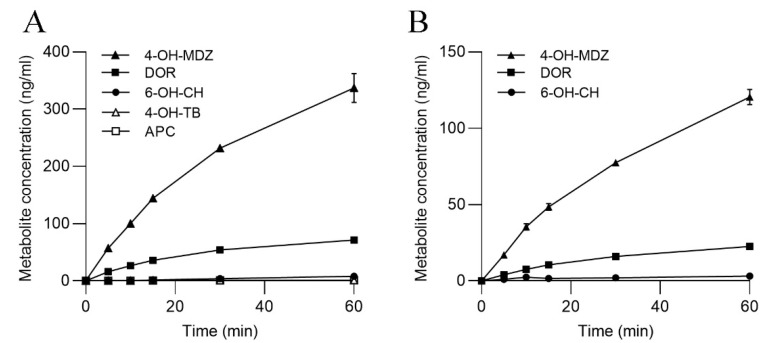
Formation of CYP-specific metabolites in (**A**) SLMs and (**B**) S9. The data represent the means ± SD of two independent experiments.

The highest conversion rate in both SLMs and S9 was achieved for MDZ, the substrate of human CYP3A4, and its piscine ortholog CYP3A27 [23] (Figure 4). Moreover, the formation of DOR, produced by human CYP2D6 or salmon CYP2K1, was notably high. 6-OH-CH formation, which is catalyzed by CYP2E1 in humans and possibly by CYP2F3 in salmon (protein existence inferred by homology in UniProt.org), was low. However, CYP1A1-produced APC was not found at a detectable level, and the same was true for 4-OH-TB, the product of CYP2C9 activity in humans and CYP2M1 in fish. 

Comparison to the results obtained in the gill cells showed differences in the tissue-specific enzyme activity profiles. While formation of 4-OH-MDZ and DOR was observed in ASG-10 and both hepatic fractions, APC reached a high level in gill cells but was only minimally produced in SLMs and S9 (Figure 2 and Figure 4). The same distribution of CYP enzyme capacities had been previously observed in a study characterizing phase I and phase II enzymes in salmon liver microsomes and S9 [18], confirming that in fish liver, CYP3A4-like activity, i.e., piscine CYP3A27, is prevalent, whereas CYP1A1 predominantly occurs in the gills [3]. However, CYP1A1 can be induced in salmon liver from BNF exposure [44], and CYP1A1, CYP2K1 (comparable to human CYP2D6) and CYP3A27 activities were dose-dependently reduced in the liver of juvenile Atlantic salmon through exposure to the environmental estrogen 4-nonylphenol [45]. 

### 3.4. UGT Activities in SLMs and S9

**Figure 5:**
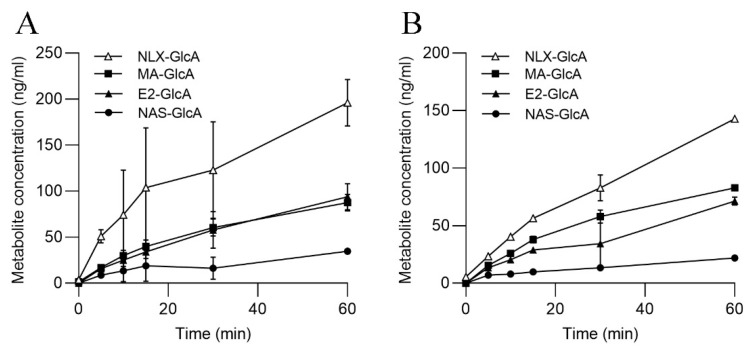
Formation of UGT-specific metabolites in (**A**) SLMs and (**B**) S9. The data represent the means ± SD of two independent experiments.

The glucuronidation capacities of SLMs and S9 were explored by using specific UGT substrates (Table 1). The highest metabolite level was reached for NLX-GlcA, a product of UGT2B7 metabolism in humans (Figure 5). The salmon orthologs that were inferred from the genome, however, have not been designated yet, though in zebrafish, homologous proteins belong to the UGT2A and UGT2B families (UniProt.org; accessed on 15 March 2023). E2-GlcA and MA-GlcA were both produced in SLMs and S9 at considerable levels. In humans, their formation indicates UGT1A1 and UGT1A9 activities, respectively, although the production of these metabolites in fish is mainly attributed to UGT5E1 and UGT1A3 catalysis [39]. We observed only low NAS-GlcA production by UGT1A6 in SLMs and S9, whereas TFP glucuronidation was not measurable (Figure 5).

The UGT activity pattern determined in the hepatic fractions in this study was comparable to previous results [18]. It also demonstrated the tissue-specific expression of fish UGTs since the profiles in SLMs and S9 (Figure 5) differed from that in ASG-10 (Figure 3), where MA-GlcA was the prevalent glucuronidation product. From this diversity, the necessity of having a suitable model for xenobiotic biotransformation in gills is evident, and ASG-10 appears to fit the role. 

### 3.5. BZ Metabolism in ASG-10

The applicability of the ASG-10 model was subsequently tested by studying the metabolism of the fish anesthetic BZ. The drug has been widely used in fish studies and in aquaculture to sedate fish and to reduce mortality during transport and stocking operations [7]. The compound can be administered by bath exposure, usually in salt form as hydrochloride to ensure solubility. Typical doses range from 10 mg/mL for sedation to 33 mg/L to 125 mg/L for anesthesia and 200 mg/L for euthanization [46]. The water solubility of non-salt BZ is only 0.4 mg/mL at 20 °C but is almost 1000 times higher in more lipidic solvents such as MeOH and chloroform. However, the n-octanol/water coefficient (pK_OW_) of 1.44 is considerably low compared to those of others, even more lipophilic anesthetics [6]. The biotransformation and pharmacokinetics of BZ have been determined in vivo in different fish species, including rainbow trout and Atlantic salmon [8,47,48]. The elimination half-lives ranged from 31 min to 89 min depending on the route of administration, applied dose and water temperature. The mean residence times were below 15 min although the distribution volumes were rather high, indicating effective metabolism and rapid excretion from the body. 

**Figure 6:**
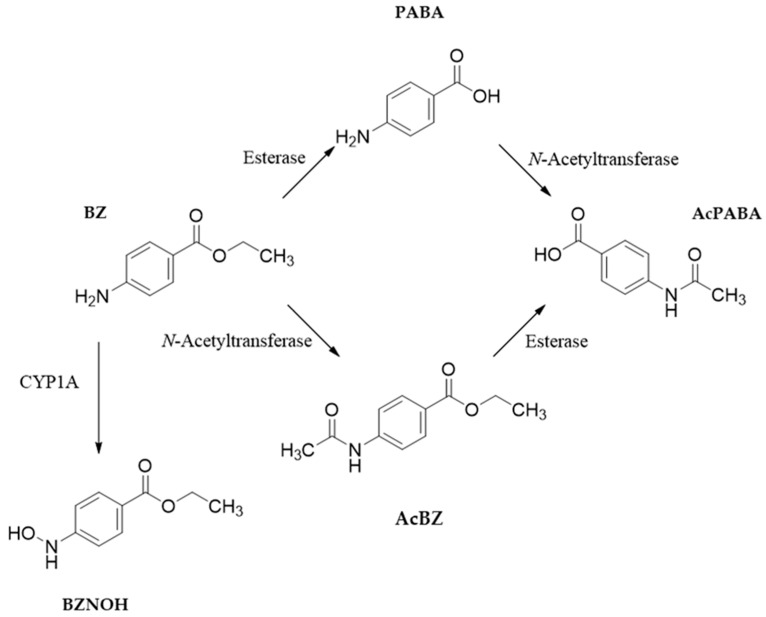
Biotransformation pathways and metabolites of benzocaine (BZ). PABA: *p*-aminobenzoic acid; AcPABA: *p*-acetaminobenzoic acid; AcBZ: acetylbenzocaine; BZNOH: benzocaine hydroxylamine.

The biotransformation of BZ has been investigated in vivo in salmon, rainbow trout and channel catfish [7,49]. BZ is metabolized by N-acetyltransferases and esterases to acetylbenzocaine (AcBZ), *p*-aminobenzoic acid (PABA) and *p*-acetaminobenzoic acid (AcPABA) (Figure 6). The major route of elimination is branchial through the gills, while renal and biliary pathways are less important.

Worryingly, it has been observed in three salmonid species that immersion in a sedating BZ dose or repeated anesthesia with BZ leads to increased blood levels of methemoglobin, the ferric iron-containing form of hemoglobin that cannot bind oxygen [50]. In fish, the most common cause of methemoglobinemia is oxidation by nitrite-containing substances such as N-phenylhydroxylamines [51]. After passive diffusion into the erythrocytes, a reaction with molecular oxygen leads to the formation of nitrosobenzene and hydrogen peroxide, which then oxidizes hemoglobin to methemoglobin. Relevantly, it has been found in a study using human hepatic S9 [52] that BZ can be converted by CYP metabolism to benzocaine hydroxylamine (BZNOH) (Figure 6), explaining its role in methemoglobin formation. Further experiments with recombinant human CYP proteins have indicated CYP1A2 as the most likely enzyme catalyzing the production of BZNOH. 

Considering the wide use of BZ in fish studies, its biotransformation by multiple pathways including the involvement of CYP1A, and the importance of the gills for BZ elimination, we selected this drug as a model compound for an in-depth investigation of the ASG-10 model’s capabilities. So far, BZ metabolism has not been studied in any fish in vitro system. 

**Figure 7:**
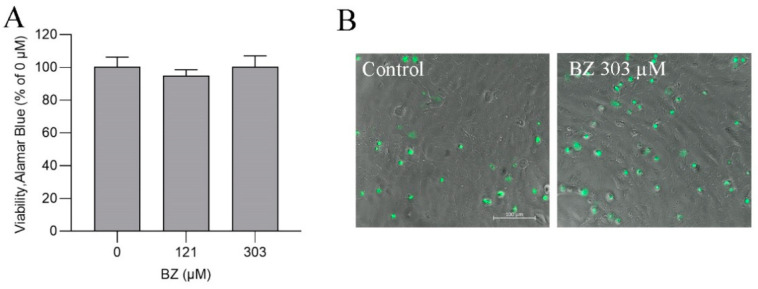
(**A**) Viability and metabolic activity determined in the Alamar Blue assay and (**B**) cytotoxicity assessed in the Celltox Green assay in ASG-10 exposed to benzocaine (BZ) for 24 h and 48 h, respectively. The data represent the means ± SD of three independent experiments, each with three technical replicates. No significance differences (*p* < 0.05) between the groups were found.

The gill cells were exposed at 121 or 303 µM BZ (Table 1), which did not affect their metabolic activity or viability, as measured in the Alamar Blue assay after 24 h (Figure 7A), and caused no cytotoxicic effects, as assessed in the Celltox Green assay (Figure 7B).

### 3.6. Determination of BZ Metabolites

**Figure 8:**
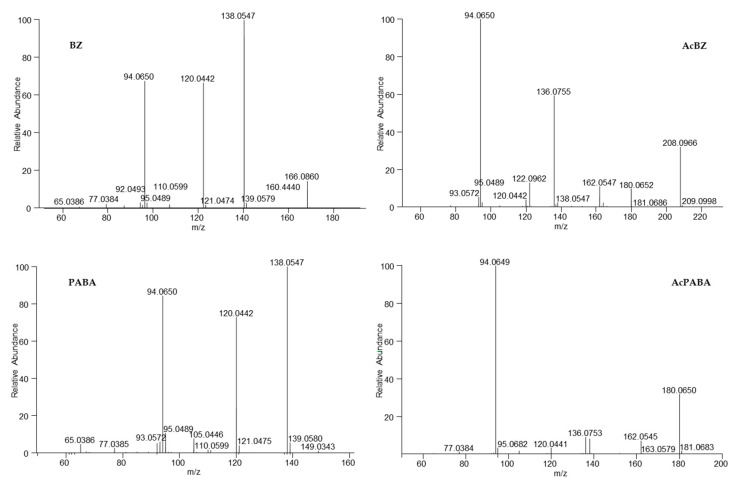
Reference standard HRMS/MS spectra ([M + H]^+^ ions) of benzocaine (BZ; *m*/*z* 166.0860), acetylbenzocaine (AcBz; *m*/*z* 208.0966), *p*-aminobenzoic acid (PABA; *m*/*z* 138.0547) and *p*-acetaminobenzoic acid (AcPABA; *m*/*z* 180.0650).

The ASG-10 cells were incubated with BZ (Table 1), and untargeted LC-HRMS/MS analysis was conducted to explore the production of BZ-related metabolites. Known BZ metabolites were defined based on comparison to the retention times and MS/MS spectra of AcBZ, PABA and AcPABA reference standards (Figure 8).

Potential BZ metabolites, for which reference standards are unavailable, were discovered by comparing the mass spectra of BZ incubations in ASG-10 to those of negative controls to find peaks of interest and by matching their mass differences and fragment ions with those measured for BZ and known metabolites.

**Figure 9:**
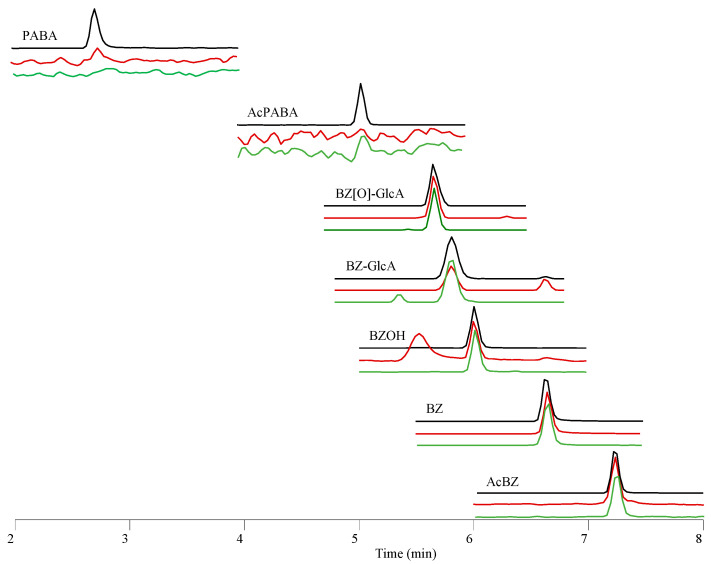
Representative extracted ion chromatograms of PRM analysis (signal abundances not normalized for better recognizability). Benzocaine (BZ) and BZ metabolites determined in plasma samples (black line) of salmon exposed to 200 mg/L BZ, ASG-10 (red line) incubated with 303 µM BZ for 24 h and hepatic S9 (green line) incubated with 1 µM BZ for 1 h.

Subsequently, the BZ metabolite profiles determined for ASG-10 were aligned (Figure 9) to results obtained from additional metabolism experiments performed for BZ in liver S9 (Table 1). Moreover, BZ metabolites were detected in the plasma of Atlantic salmon that had been euthanized with a BZ overdose. The extracted LC-HRMS ion chromatograms showed strong similarities between the metabolites formed in ASG-10, S9 and in vivo, with the exception of PABA and AcPABA, which occurred at measurable levels only in the salmon plasma (Figure 9).

In total, six BZ metabolites were determined in the different samples. Besides the previously described AcBZ, PABA and AcPABA (Table 3), we additionally discovered a hydroxylated metabolite BZOH, possibly BZNOH, as well as glucuronidation products of both BZ and BZOH. 

In the few reports published on BZ metabolites in fish in vivo [7,48], only AcBZ, PABA and AcPABA were described, although studies using radioactively marked BZ indicated the potential existence of additional, unknown metabolites [53]. BZ metabolites were measured in different tissues after water exposure or intra-arterial administration to rainbow trout [7,48,53]. Interestingly, the BZ distribution and elimination kinetics were dependent on water temperature, with 6 °C resulting in prolonged BZ half-life and protein binding in plasma than 12 °C or 18 °C, as well as lower clearance. Accordingly, the plasma concentration–time profiles of BZ, AcBZ, PABA and AcPABA increased with colder temperatures. In channel catfish [49], BZ, AcBZ and AcPABA were detected in most tissues for up to 144 h after water exposure to BZ, whereas PABA was detected only in the liver and bile. Gender differences were not observed. Zebrafish embryos administered BZ in water [54] produced AcBZ and PABA, which accounted for 90% of the total BZ amount after 48 h exposure. 

**Figure 10:**
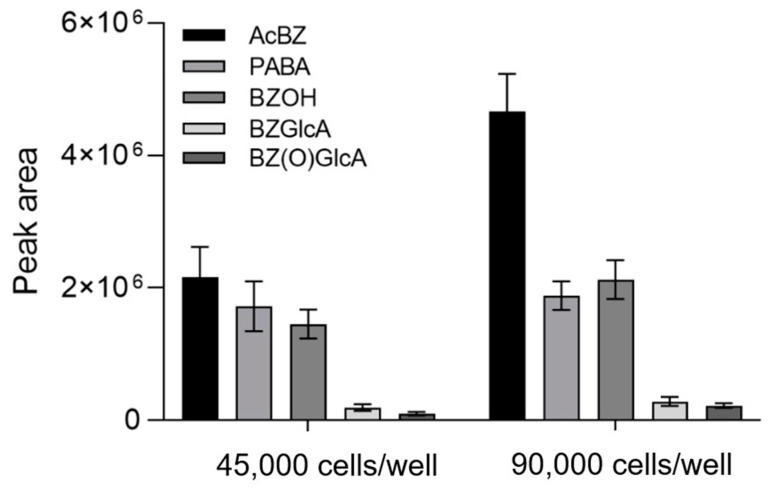
Benzocaine (BZ) metabolites produced in ASG-10 cells (45,000 or 90,000 cells/well) during 24 h incubation (at 303 µM). Levels are shown as measured LC-HRMS peak areas. Data represent means ± SD of three technical replicates.

The BZ metabolites discovered in the different Atlantic salmon samples in the present study were characterized in more detail in additional experiments. AcBZ was the major biotransformation product in ASG-10 cells (Figure 10). The AcBZ levels increased in accordance with the number of cells, indicating high N-acetyltransferase activity in ASG-10 (Figure 6). The metabolite was determined by comparison of the retention time and LC-HRMS/MS fragment data (Supplementary Figure S1) to those of the reference standard (Figure 8). The congruency of the specific fragments at *m*/*z* 94.0650 (C_6_H_8_N^+^; ∆ ± 5 ppm) and *m*/*z* 136.0755 (C_8_H_10_NO^+^; ∆ ± 5 ppm) allowed for unambiguous establishment of AcBZ. The prevalence of AcBZ in the ASG-10 incubations reflected the in vivo situation well [55], where about 59% of the applied BZ dose in rainbow trout was eliminated, through the gills within 3 h, mainly as AcBZ.

The protonated molecular ion at *m*/*z* 138.0547 (C_7_H_8_NO_2_^+^; ∆ ± 5 ppm) in the full-scan mass spectrum was determined as PABA (Figure 9) based on the similar retention time to the reference standard and the exact mass (Supplementary Figure S2). However, insufficient fragmentation due to low signal intensity and considerable background noise from the matrix during the early stages of retention (2.7 min; Table 3) meant higher uncertainty regarding the determination of this metabolite. Nevertheless, the typical PABA fragments (*m*/*z* 94.0659 and *m*/*z* 120.0442) were found in the plasma samples, confirming the formation of this metabolite in salmon. In ASG-10 cells, the production of PABA was only slightly increased with higher cell density (Figure 10). Moreover, the metabolite was not detectable in SLM and S9 incubations, which is consistent with PABA being formed by plasma esterase activity (Figure 6). Consequently, AcPABA was also only detected in the plasma samples (Figure 9). PABA is a non-protein amino acid of physiological significance as a folate precursor and immunomodulator and is part of the vitamin B complex [56]. The levels in plasma and cells are thus likely subject to considerable fluctuations depending on the capacities of subsequent enzymatic reactions. Conjugation products of PABA that are known from studies in humans, such as *p*-aminohippuric acid formed with glycine [57] or PABA-glucuronide [58,59], were not detected.

The peak with the retention time of 6.0 min (*m*/*z* 182.0811; [M + H]^+^) in the full-scan chromatograms of Atlantic salmon plasma, ASG-10 and S9 (Figure 9) had a theoretical mass increase of +16 Da as compared to BZ, indicating oxygen addition as a result of hydroxylation (Table 3). The hydroxylated metabolite BZOH demonstrated the typical fragmentation pattern in the product ion spectrum (Supplementary Figure S3) that is observed for BZ, though with additional oxygen in several fragments (Table 4; Supplementary Figure S4). 

**Figure 11:**
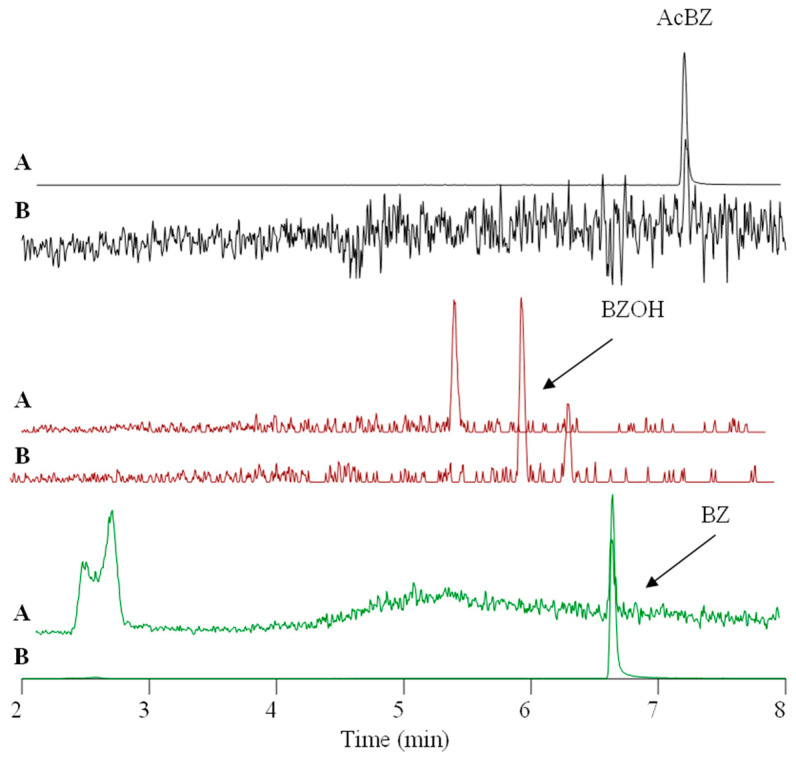
Extracted ion chromatograms showing a comparison of the hydroxylation efficiencies in SLMs after 1 h incubation of (**A**) AcBZ and (**B**) BZ. The metabolite BZOH is not formed when AcBZ is used as a precursor because the amino group is blocked by the acetylation.

The product ion spectrum of BZOH was dominated by the ion at *m*/*z* 154.0497 (C_7_H_8_O_3_N^+^), which was congruent to the fragment of a metabolite that had been previously observed by MS/MS analysis after incubation of BZ with recombinant CYP1A2 and identified as N-hydroxylated BZNOH [52]. Aiming to narrow down the site of hydroxylation in the BZOH that we had produced in salmon ASG-10 and liver fractions, we used N-acetylated BZ (AcBZ) as the starting substance in an incubation in SLMs. Since a hydroxylation product was no longer detectable under these conditions because the amino group of BZ was blocked by the acetylation (Figure 11), we concluded that the hydroxylated metabolite determined in the salmon samples was in fact also BZNOH.

In addition to the metabolites formed by phase I enzyme reactivity, we discovered two phase II glucuronidation products in the different salmon samples, which eluted at 5.6 min and 5.8 min in LC-HRMS analysis (Figure 9; Table 3). The latter, at *m*/*z* 342.1180 ([C_15_H_20_NO_8_^+^, ∆ ± 5 ppm), was fragmented in MS/MS to the product ions at *m*/*z* 166.0861 (C_9_H_12_NO_2_ ^+^; ∆ ± 5 ppm), featuring the characteristic neutral loss of 176 Da (C_6_H_8_O_6_, ∆ < 2 ppm) for a glucuronide, and *m*/*z* 138.0548 (C_7_H_8_NO_2_^+^; ∆ ± 5 ppm) (Supplementary Figure S5). The metabolite was thus established as a glucuronide of BZ (BZGlcA) (Table 3). Its level in ASG-10 increased slightly with higher cell density (Figure 10). The metabolite was absent in control samples without added BZ, confirming its origin. BZGlcA is very likely the product of N-glucuronidation in the BZ amino group, corresponding to the PABA-N-glucuronide and AcPABA-N-glucuronide detected in human urine after PABA exposure [58]. The existence of BZGlcA was predicted in exploratory studies on BZ metabolism in rabbits and rats [60,61] but had not been confirmed yet. The characterization of BZGlcA by LC-HRMS/MS in the present study, providing the exact mass data and the fragmentation pattern, is thus the first detailed description of a BZ glucuronidation metabolite. 

The second conjugated metabolite that we discovered in the salmon samples (Figure 9; Table 3) was defined as a glucuronide of BZNOH (BZ(O)GLcA) at *m*/*z* 358.1136 (C_15_H_20_NO_9_^+^_;_ ∆ ± 5 ppm; +176 Da addition to BZOH). The MS/MS analysis yielded several product ions, including at *m*/*z* 182.0811 (C_9_H_12_NO_3_^+^; ∆ ± 5 ppm; loss of 176 Da) and *m*/*z* 154.0497 (C_7_H_8_NO_3_^+^; ∆ ± 5 ppm) (Supplementary Figure S6), which are characteristic for BZOH (Table 4; Supplementary Figures S3 and S6). In ASG-10 cells, BZ(O)GLcA levels showed dependency on cell density (Figure 10). The metabolite was not produced in the absence of BZ. Glucuronidated BZOH has not been previously reported; however, *in vivo* metabolism of dimethocaine (DMC) [62] (a structural analog of BZ) in rats resulted in the formation of, among others, DMC(O)GlcA and DMCGlcA. Interestingly, N-glucuronidation of primary aromatic amines such as BZ and DMC is catalyzed in humans by enzymes such as UGT1A1, UGT1A9 and UGT2B7 [63]. Since we were able to establish UGT1A9-like and UGT2B7-like enzyme activities in the present study in ASG-10 (Figure 3) and the liver fraction S9 (Figure 5), respectively, we presumed that salmon also has the capacity to glucuronidate aromatic hydroxylamines, explaining the presence of BZ(O)GlcA in the gill cells, liver fraction and plasma (Figure 9). 

## 4. Conclusions

The ASG-10 gill cell line from Atlantic salmon is a competent model for in vitro biotransformation studies of xenobiotics. The activities of CYP and UGT prevalent in fish gills were demonstrated in functional assays with specific substrates. The metabolic enzyme profiles differed to those in hepatic fractions of salmon, showing the importance of the correct choice of model with respect to the actual research question. ASG-10 contained inducible CYP1A1, comparable to the in vivo situation. Furthermore, metabolism experiments with the fish anesthetic BZ confirmed esterase and N-acetyltransferase in the gill cells and led to the determination of BZOH and two glucuronidated metabolites, BZGlcA and BZ(O)GlcA, which have not been described in fish previously. 

## Figures and Tables

**Table 1:** Substrate and metabolite concentrations in the different in vitro experiments.

				Combined Substrate Solutions [µM]				Working Solutions [mM]		Assay Concentrations [µM]	
				C1	C2	C3	C4	W5	W6 and W7		
Activity	Substrate Product	Compound	Stock Solution [mM]	ASG-10	SLM/S9	ASG-10	SLM/S9	ASG-10	SLM/S9	ASG-10	SLM/S9
CYP1A2	PCN	phenacetin	6.1	1000	715					2; 5; 10	2.5
	ACP	acetaminophen	7.3								
CYP2C9	TB	tolbutamide	3.7	1000	710					2; 5; 10	2.5
	4-OH-TB	4-hydroxy-TB	3.5								
CYP2D6	DEX	dextromethorphan	3.7	1000	715					2; 5; 10	2.5
	DOR	dextrophan	3.9								
CYP2E1	CH	chlorzoxazone	5.9	1000	1444					2; 5; 10	5.1
	6-OH-CH	6-hydroxy-CH	5.4								
CYP3A4	MDZ	midazolam	3.1	1000	2824					2; 5; 10	10
	4-OH-MDZ	4-hydroxy-MDZ	2.9								
UGT1A1	E2	β-estradiol	3.7			1000	250			2; 5; 10	5.5
	E2-GlcA	E2-17β-D-glucuronide	2.1								
UGT1A4	TFP	trifluoperazine 2HCl	2.5			1000	83			2; 5; 10	1.2
	TFP-GlcA	TFP-N-β-D-glucuronide	0.2								
UGT1A6	NAS	N-acetylserotonin	46			1000	268			2; 5; 10	6.9
	NAS-GlcA	NAS-β-D-glucuronide	2.5								
UGT1A9	MA	mycophenolic acid	12			1000	83			2; 5; 10	0.5
	MA-GlcA	MA-β-D-glucuronide	2.0								
UGT2B7	NLX	naloxone	3.1			1000	168			2; 5; 10	3.1
	NLX-3GlcA	NLX-3β-D-glucuronide	2.0								
NAT and esterases	BZ	benzocaine	6.1					121	0.4; 1.21	121; 303	1; 3
	AcPABA	*p*-acetaminobenzoic acid	5.6								
	PABA	*p*-aminobenzoic acid	7.3								
	AcBZ	acetylbenzocaine	4.8								

Cytochrome P450 (CYP); glucuronosyltransferases (UGT); N-acetyltransferases (NAT). SLM/S9: concentrations given in the respective columns were applied in incubation experiments with SLM or S9 salmon liver fractions.

**Table 2:** Cofactor concentrations in SLM and S9 biotransformation assays.

Concentration [mM]	SLM	S9
NADPH	0.95	0.92
NADP+	0.87	0.84
Glucose 6-phosphate	20.3	19.6
MgCl_2_ × 6H_2_O	9.43	9.13

**Table 3:** Retention times, accurate and observed masses of LC-HRMS, mass error and composition of BZ and BZ metabolites detected in ASG-10, S9 and salmon plasma ^#^.

Metabolite		Composition	Theoretical Mass [M + H]^+^ *m*/*z* [Da]	Observed Mass [M + H]^+^ *m*/*z* [Da]	Mass Error *∆ ppm	Retention Time [min]
*p*-Aminobenzoic acid	PABA	C_7_H_7_NO_2_	138.0550	138.0547	−1.775	2.7
*p*-Acetaminobenzoic acid	AcPABA	C_9_H_9_NO_3_	180.0655	180.0650	−2.609	5.0
Benzocaine hydroxylamine glucuronide	BZ(O)GlcA	C_15_H_19_NO_9_	358.1133	358.1136	0.984	5.6
Benzocaine glucuronide	BZGlcA	C_15_H_19_NO_8_	342.1183	342.1180	−0.856	5.8
Benzocaine hydroxylamine	BZOH	C_9_H_11_NO_3_	182.0812	182.0811	−0.438	6.0
Benzocaine	BZ	C_9_H_11_NO_2_	166.0863	166.0860	−1.356	6.6
Acetylbenzocaine	AcBZ	C_11_H_13_NO_3_	208.0968	208.0967	−0.768	7.2

* Mass errors (<±5 ppm) calculated for observed mass to theoretical mass from LC-HRMS/MS spectra (Supplementary Figures S1–S3, S5 and S6). ^#^ Distribution of the different BZ metabolites in the different samples is shown in Figure 9.

**Table 4:** Observed exact masses and calculated molecular compositions of the major fragment ions in the LC-HRMS/MS spectra of the protonated molecular ions [M + H]^+^ of BZ and the hydroxylated metabolite BZOH.

BZ			BZOH		
*m*/*z* [Da]	Composition	Mass Error * [ppm]	*m/z* [Da]	Composition	Mass Error * [ppm]
166.0860	C_9_H_12_O_2_N	−1.657	182.0811	C_9_H_12_O_3_N	−0.328
138.0547	C_7_H_8_O_2_N	−1.92	154.0497	C_7_H_8_O_3_N	−1.166
120.0442	C_7_H_6_ON	−1.669	136.0392	C_7_H_6_O2N	−0.478
94.0650	C_6_H_8_N	−1.657	110.0599	C_6_H_8_ON	−1.094

* Mass errors (<±5 ppm) calculated for observed mass to theoretical mass from LC-HRMS/MS spectra (Figure 8; Supplementary Figure S3).

## Data Availability

The data presented in this study are available from the corresponding author on reasonable request due to privacy.

## Supplementary Materials

The following supporting information can be downloaded at: https://www.mdpi.com/article/10.3390/metabo13060771/s1, Table S1: Calibration data of the UHPLC TQMS method for liver microsomes from Atlantic salmon; Table S2: Calibration data of the UHPLC TQMS method for ASG-10 cells from Atlantic salmon; Figure S1: Positive ion LC-HRMS/MS spectra of AcBZ; Figure S2: Positive ion LC-HRMS/MS spectra of PABA; Figure S3: Positive ion LC-HRMS/MS spectra of BZOH; Figure S4: Tentative fragmentation patterns of BZ and BZOH; Figure S5: Positive ion LC-HRMS/MS spectra of BZGLcA; Figure S6: Positive ion LC-HRMS/MS spectra of BZ(O)GLcA.
