# Supplementary material for: Atlantic Salmon Gill Epithelial Cell Line (ASG-10) as a Suitable Model for Xenobiotic Biotransformation
Source: Metabolites. 2023 Jun 20;13(6):771. doi: 10.3390/metabo13060771 (PMC10303838; doi:10.3390/metabo13060771)
Supplement: Supplementary file 1 [file metabolites-13-00771-s001.zip › metabolites-2421556-supplementary.pdf]

Article

Atlantic Salmon Gill Epithelial Cell Line (ASG-10) as a Suitable Model for Xenobiotic Biotransformation Lada Ivanova [1]*, Christiane Kruse Fæste [1] and Anita Solhaug [1]

Supplementary Table S1. Performance parameters of the LC–TQMS method applied for the semi-quantitative analysis of respective metabolites of several CYP and UGT probe substrates in liver microsomes from Atlantic salmon.

	ACP	6-OH-CH	4-OH-TB	DOR	4-OH-MDZ	NLX-GlcA	E2-GlcA	NAS-GlcA	TFP-GlcA	MA-GlcA
Linear range [ng/ml]*	7.5–321	7.5–300	7.5–300	7.5–300	7.5–300	6.3–250	6.3–250	6.3–250	6.3–250	6.3–250
Correlation coefficient [R^2]*	0.999	0.9998	0.9997	1.0000	0.9999	0.9927	0.9986	0.9935	0.9937	0.9986
LOD* [ng/ml]	9.9	4.0	4.9	1.4	2.4	18.6	8.2	17.6	17.4	8.1
LOQ* [ng/ml]	33	13	16	4.6	8.0	62	27	59	58	27
SSE%	75	50	122	92	136	203	100	130	129	92
RSD [%]+	<LOD	37	<LOD	43	18	12	11	56	<LOD	17

*The matrix-assisted calibration standards were used as the basis for this analysis, with 2 to 4 independent experiments conducted; +the metabolites produced were detected in 60 min-incubation mixtures at levels above the limit of detection (LOD), with 5 or 6 replicates utilized.

Supplementary Table S2. Performance parameters of the LC–TQMS method applied for the semi-quantitative analysis of respective metabolites of several CYP and UGT probe substrates in ASG-10 cells from Atlantic salmon.

	ACP	6-OH-CH	4-OH-TB	DOR	4-OH-MDZ	NLX-GlcA	E2-GlcA	NAS-GlcA	TFP-GlcA	MA-GlcA
Linear range [ng/ml]*	7.5–321	7.5–300	7.5–300	7.5–300	7.5–300	6.3–250	6.3–250	6.3–250	6.3–250	6.3–250
Calibration coefficient [R^2]*	0.9985	0.9993	0.9999	0.9999	0.9985	0.9998	0.9995	0.9999	0.9992	0.9991
LOD* [ng/ml]	11.0	6.8	3.1	2.1	10.5	3.0	4.6	2.6	6.0	6.5
LOQ* [ng/ml]	37	23	10	6.9	34.9	10	15	9	20	22
SSE%	63	42	98	96	91	105	88	102	128	102
RSD [%]+1	43-71	<LOD	<LOD	1–3	5–10	<LOD	21–30	7–11	<LOD	22–29

*The matrix-assisted calibration standards were used as the basis for this analysis, with 3 independent experiments conducted; +Metabolites were identified following 24 hours of incubation with varying concentrations of CYP and UGT substrates (2 µM, 5 µM, and 10 µM) at levels above the limit of detection (LOD). For each concentration, three replicates were utilized… [1]Data are presented as a range min–max.

Supplementary Figures

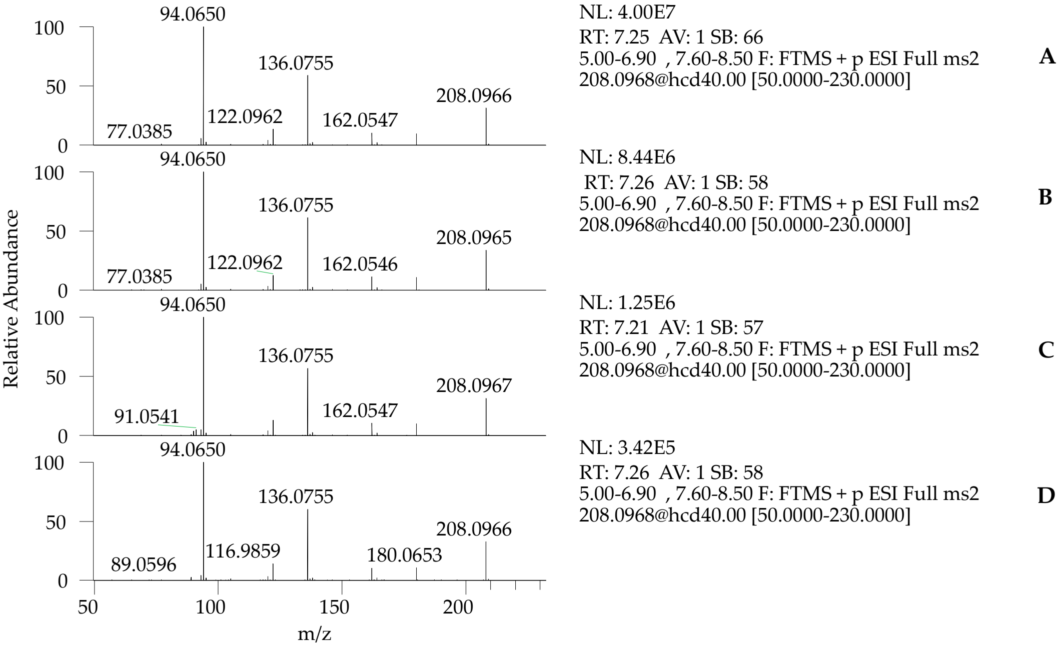

Figure S1. Positive ion LC-HRMS/MS spectra of acetylbenzocaine (AcBZ, $[M+H]^+$; theoretical mass m/z 208.0968) in (A) reference standard, (B) salmon plasma, (C) incubation medium of ASG-10 cells after 24 h exposure to 303 μM benzocaine (BZ), and (D) S9 incubation aliquot (4 mg/ml protein, 1 μM BZ, 1 h).

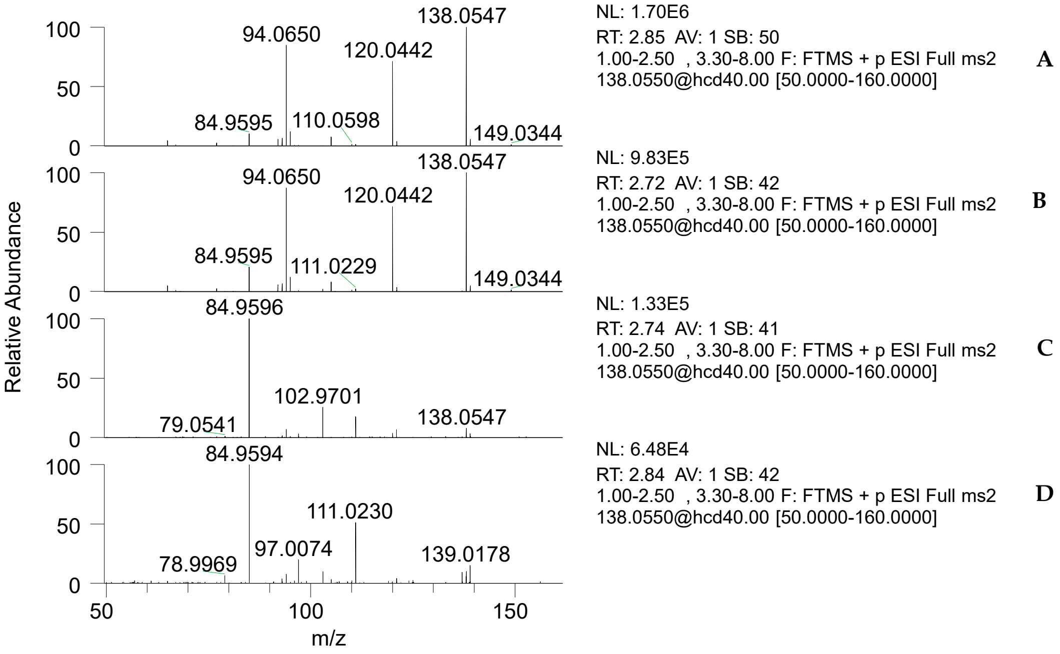

Figure S2. Positive ion LC-HRMS/MS spectra of p-aminobenzoic acid (PABA, $[M+H]^+$; theoretical mass *m/z* 138.0550) in (A) reference standard, (B) salmon plasma, (C) incubation medium of ASG-10 cells after 24 h exposure to 303 μM benzocaine (BZ) (C), and (D) S9 incubation aliquot (4 mg/ml protein, 1 μM BZ; 1h).

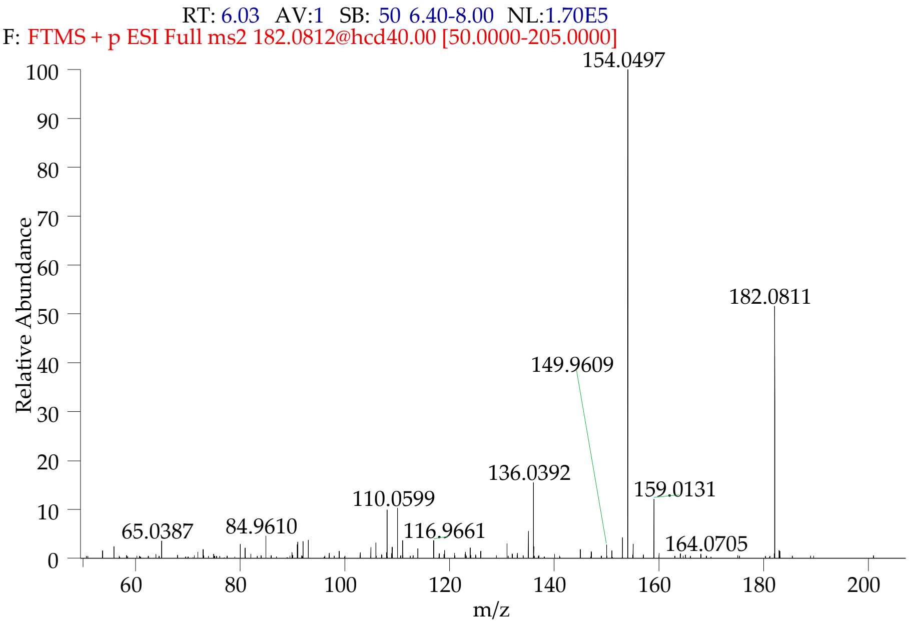

Figure S3. Positive ion LC-HRMS/MS spectra of benzocaine hydroxylamine (BZOH, $[M+H]^+$, theoretical mass *m/z* 182.0812) detected in incubation medium of ASG-10 cells after 24 h exposure to 303 μM benzocaine (BZ).

A

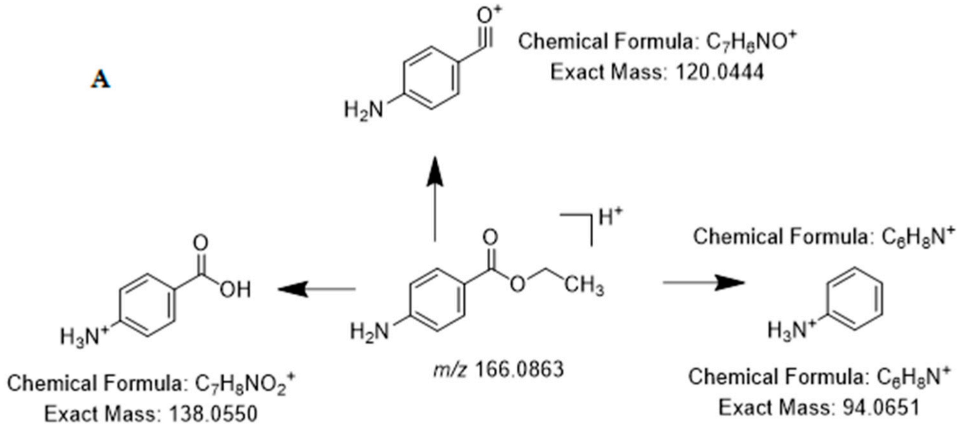

B

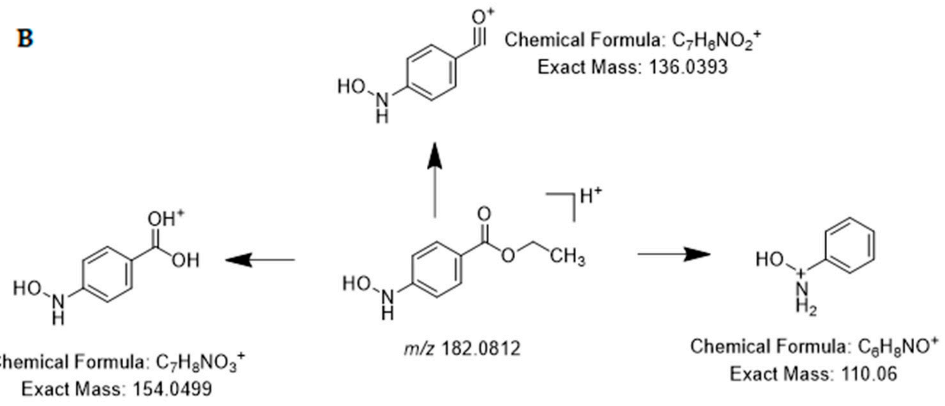

Figure S4. Tentative fragmentation patterns of **A)** BZ and **B)** BZOH in LC-HRMS/MS. BZ: benzocaine; BZOH: benzocaine hydroxylamine.

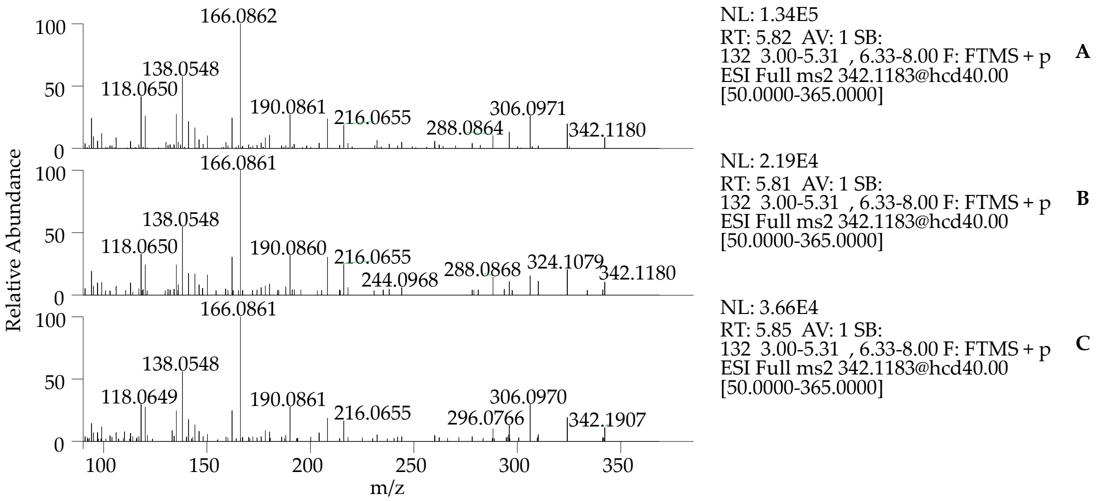

Figure S5. Positive ion LC-HRMS/MS spectra of benzocaine glucuronide (BZGlcA, $[M+H]^+$; theoretical mass *m/z* 342.1183) in (A) salmon plasma, (B) incubation medium of ASG-10 cells after 24 h exposure to 303 µM benzocaine (BZ), and (C) S9 incubation aliquot (4 mg/mL protein, 1 µM BZ; 1h). The ions at *m/z* 166.0861 and *m/z* 166.0862 ($C_9H_{12}O_2N$, $\Delta < 1$ ppm) were formed by neutral loss of 176 Da ($C_6H_8O_6$, $\Delta < 2$ ppm), which is a characteristic feature of a glucuronide.

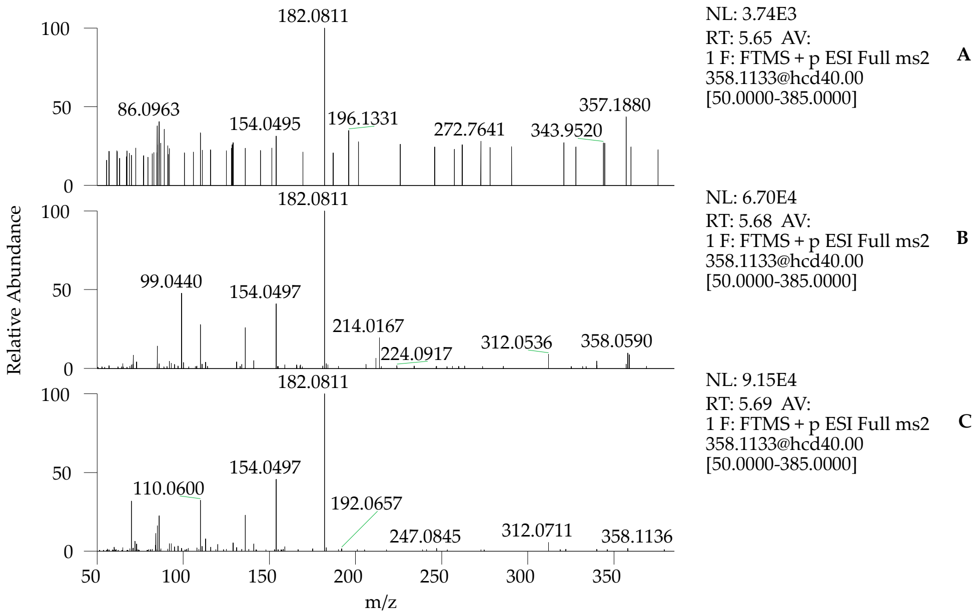

Figure S6. Positive ion LC-HRMS/MS spectra of benzocaine hydroxylamine glucuronide (BZ(O)GlcA, $[M+H]^+$; theoretical mass *m/z* 358.1133) in (A) salmon plasma, (B) incubation medium of ASG-10 cells after 24 h exposure to 303 µM benzocaine (BZ), and (C) S9 incubation aliquot (4 mg/mL protein, 1 µM BZ; 1h). The ions at *m/z* 182.0811 ($C_9H_{12}O_3N$, $\Delta < 1$ ppm) were generated through the neutral loss of 176 Da ($C_6H_8O_6$, $\Delta = 2.2$ ppm), which is a characteristic feature of a glucuronide.
